# Draft Genome Sequence of the Yeast *Rhodotorula* sp. Strain CCFEE 5036, Isolated from McMurdo Dry Valleys, Antarctica

**DOI:** 10.1128/MRA.00020-20

**Published:** 2020-04-02

**Authors:** Claudia Coleine, Sawyer Masonjones, Silvano Onofri, Laura Selbmann, Jason E. Stajich

**Affiliations:** a Department of Ecological and Biological Sciences, University of Tuscia, Viterbo, Italy; b Department of Microbiology and Plant Pathology, University of California—Riverside, Riverside, California, USA; c Italian National Antarctic Museum (MNA), Mycological Section, Genoa, Italy; Vanderbilt University

## Abstract

A draft genome sequence was assembled and annotated of the basidiomycetous yeast *Rhodotorula* sp. strain CCFEE 5036, isolated from Antarctic soil communities. The genome assembly is 19.07 megabases and encodes 6,434 protein-coding genes. The sequence will contribute to understanding the diversity of fungi inhabiting polar regions.

## ANNOUNCEMENT

*Rhodotorula* fungi are ubiquitous saprophytic yeasts taxonomically classified in the Pucciniomycotina and Ustilaginomycotina subphyla (phylum Basidiomycota) (1, 2). These fungi can be isolated from many environments and are often found associated with humans, animals, and food (3). Species have been described from the gut microbiota of carnivorous fish (4) and contaminated soil (5). Some members of this group are cryophilic extremophiles and can persist under extreme conditions (low temperature, high salinity, high pressure, and low pH) (6–11). The genome sequence of an Antarctic *Rhodotorula* isolate will be useful for comparative studies of evolution of extremophilic yeasts, in efforts to study their role in biogeochemical nutrient cycling in cold environments, and in bioprospecting for new enzymes (12, 13).

A *Rhodotorula* sp. culture was isolated from soil collected near a glacier during the XI Italian Antarctic Expedition (1995 to 1996) at Edmonson Point at 74°20′00″S, 165°08′00″E (Northern Victoria Land, Continental Antarctica), an Antarctic Specially Protected Area (ASPA), following the protocol described by Selbmann et al. (14). Briefly, soil was sprinkled on petri dishes containing 2% malt extract agar (MEA; AppliChem GmbH, Darmstadt, Germany) supplemented with 100 ppm chloramphenicol and incubated at 10°C for several months. Yeast colonies were streaked onto fresh medium to isolate pure cultures. *Rhodotorula* sp. CCFEE 5036 strain culture is deposited in the Culture Collection of Fungi from Extreme Environments (CCFEE; University of Tuscia, Italy) and at the Dipartimento di Biologia Vegetale e Agroambientale of the University of Perugia Industrial Yeasts Collection (DBVPG) as strain 5527. Genomic DNA was extracted from a pure culture grown for 3 weeks at 10°C on MEA following the cetyltrimethylammonium bromide (CTAB) protocol (15). The DNA was sheared with a Covaris S220 ultrasonicator, and a sequencing library was constructed using the Neoprep TruSeq nano DNA sample prep protocol (Illumina, Inc., San Diego, CA) in a genomics core (Institute for Integrative Genome Biology, University of California, Riverside). The library was multiplexed and sequenced on an Illumina MiSeq flow cell to obtain 6.1 million 2 × 300-bp paired-end sequence reads. FastQC (v0.11.3) was used to check read quality (16).

Genome assembly was performed with MaSuRCA (v2.3.2) (17) using default parameters (cgwErrorRate, 0.15), which included quality-based read trimming and corrections. Trimmed reads averaged 199 bp. Assembled scaffolds were filtered for vector contamination with Sequin (v15.10) (https://www.ncbi.nlm.nih.gov/Sequin/), and redundant scaffolds were eliminated if they aligned with at least 95% identity to a longer contig with MUMMer (v3.23) (18), using the “clean” step in Funannotate (v0.5.5) (19). The assembly was 155 contigs and totaled 19.08 Mb in length (*N*_50_, 338 kb; *L*_50_, 19; longest scaffold, 930,366 bp; G+C content, 60.58%; average depth of coverage, 192×).

Genome annotation performed by Funannotate (v0.5.5) (19) produced consensus gene models by EVidenceModeler (EVM) (20), combining *ab initio* predictions from AUGUSTUS (v3.2.2) (21) and GeneMark.hmm-ES (v4.32) (22) with protein-to-genome alignments from Exonerate (v.2.2.0) (23). GeneMark.hmm-ES self-training used default parameters, and AUGUSTUS was trained with alignments of BUSCO basidiomycota_odb9 proteins (v9) (24) and gene prediction parameters archived in a GitHub repository (25). Gene functions were assigned by similarity to Pfam (26), MEROPS (27), CAZy (28, 29), eggNOG (v4.5) (30), InterProScan (31), and Swissprot (32) databases by BLASTP (v2.5.0+) or HMMER3 (33) searches using Funannotate default parameters. A total of 6,553 protein-coding genes were predicted and prepared for GenBank submission by Genome Annotation Generator (34).

### Data availability.

This whole-genome shotgun project was deposited at DDBJ/ENA/GenBank under the accession number MXAQ00000000. The version described in this paper is the first version, MXAQ01000000. Illumina sequence reads are released under SRA accession number SRR5223778 and associated with BioProject PRJNA342238.
